# Supplementary material for: Thymoquinone as an electron transfer mediator to convert Type II photosensitizers to Type I photosensitizers
Source: Nat Commun. 2024 Jun 10;15:4943. doi: 10.1038/s41467-024-49311-z (PMC11164902; doi:10.1038/s41467-024-49311-z)
Supplement: Supplementary file 1 — Supplementary Information [file 41467_2024_49311_MOESM1_ESM.pdf]

Supplementary Information

Thymoquinone as an electron transfer mediator to convert Type II photosensitizers to Type I photosensitizers

Jiahao Zhuang[1,2], Guobin Qi[2], Yecheng Feng[3], Min Wu[1], Hang Zhang[4], Dandan Wang[1,2], Xianhe Zhang[2], Kok Chan Chong[2], Bowen Li[2], Shitai Liu[1,2], Jianwu Tian[2], Yi Shan[2], Duo Mao[3]*, Bin Liu[1,2]*

[1]Joint School of National University of Singapore and Tianjin University, International Campus of Tianjin University, Binhai New City, Fuzhou 350207, China.
[2]Department of Chemical and Biomolecular Engineering, National University of Singapore, Singapore 117585, Singapore.
[3]Institute of Precision Medicine, The First Affiliated Hospital of Sun Yat-Sen University, Sun Yat-Sen University, Guangzhou 510080, China.
[4]Department of Materials Science and Engineering, National University of Singapore, Singapore 117574, Singapore.

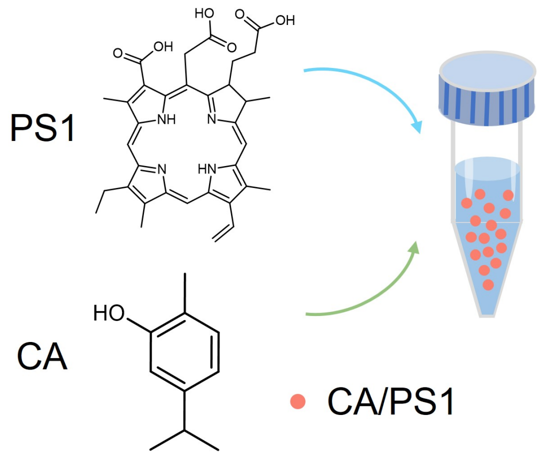

Supplementary Figure 1. Schematic illustration of the formation of CA/PS1 complex.

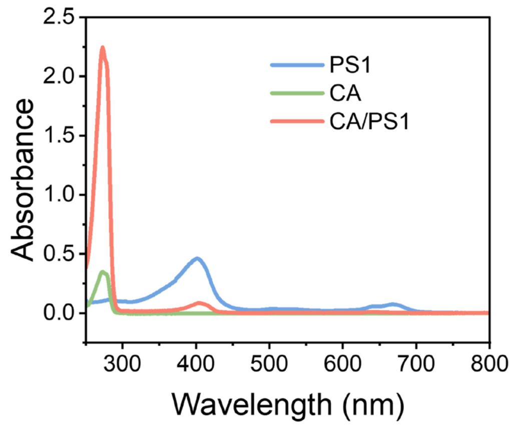

Supplementary Figure 2. UV-Vis spectra of PS1, CA, and CA/PS1. The samples were filtered through a 0.45 μm filter and then characterized by UV-Vis.

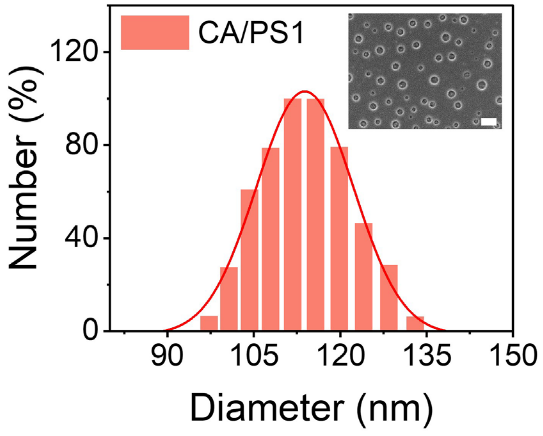

Supplementary Figure 3. Hydrodynamic particle diameter measured by DLS of CA/PS1 complex in PBS, insert is the SEM image of CA/PS1 complex, scale bar = 200 nm.

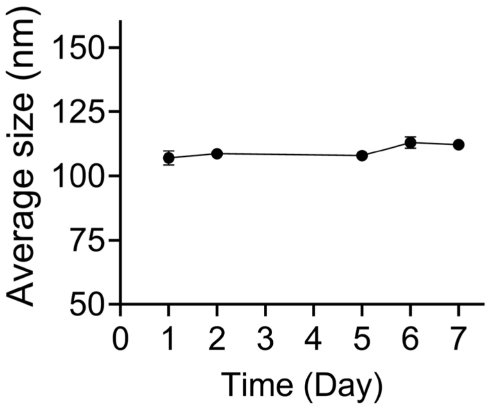

Supplementary Figure 4. Hydrodynamic particle diameter of CA/PS1 complex dispersed in PBS for different storage periods at 4 °C. Data represented as mean ± SD from n = 3 independent samples.

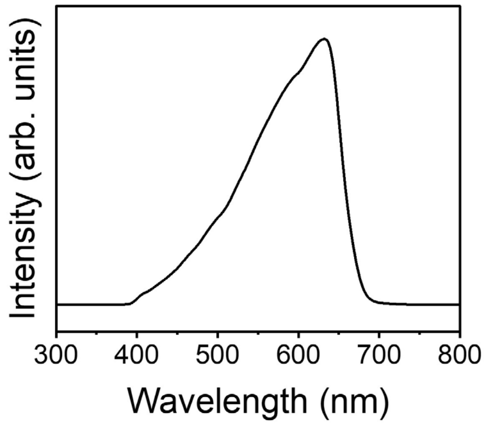

Supplementary Figure 5. Wavelength distribution of the light source.

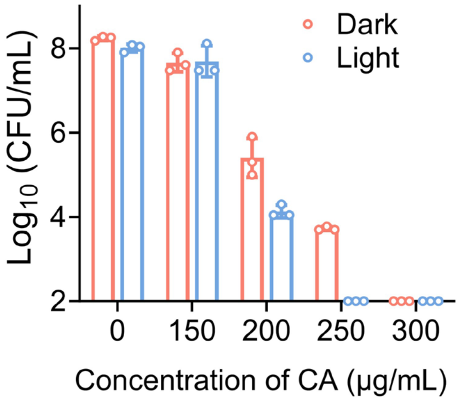

Supplementary Figure 6. CFU counts of *S. aureus* treated with different concentrations of CA (0-300 μg/mL) with or without light irradiation (60 mW/cm^2, 10 min). Data represented as mean ± SD from n = 3 independent biological samples.

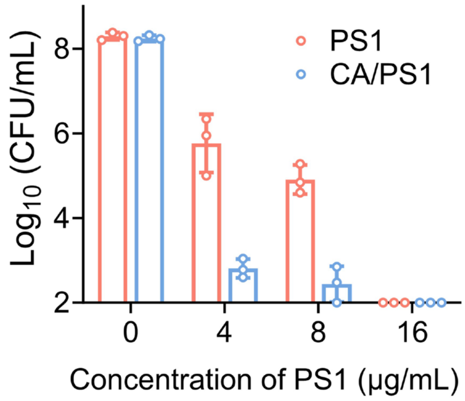

Supplementary Figure 7. CFU counts of *S. aureus* treated with different concentrations of PS1 (0-16 μg/mL) or CA/PS1 (fixed concentration of CA at 150 μg/mL, varying concentrations of PS1 from 0-16 μg/mL) upon light irradiation (60 mW/cm^2, 10 min). Data represented as mean ± SD from n = 3 independent biological samples.

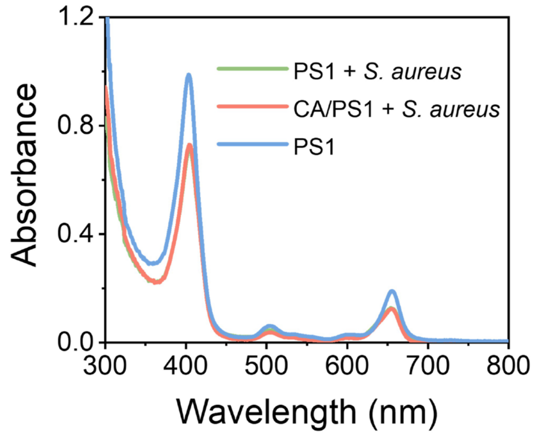

Supplementary Figure 8. Absorption of isolated PS1 from *S. aureus* culture medium when treated with or without CA (150 μg/mL) for 3 h.

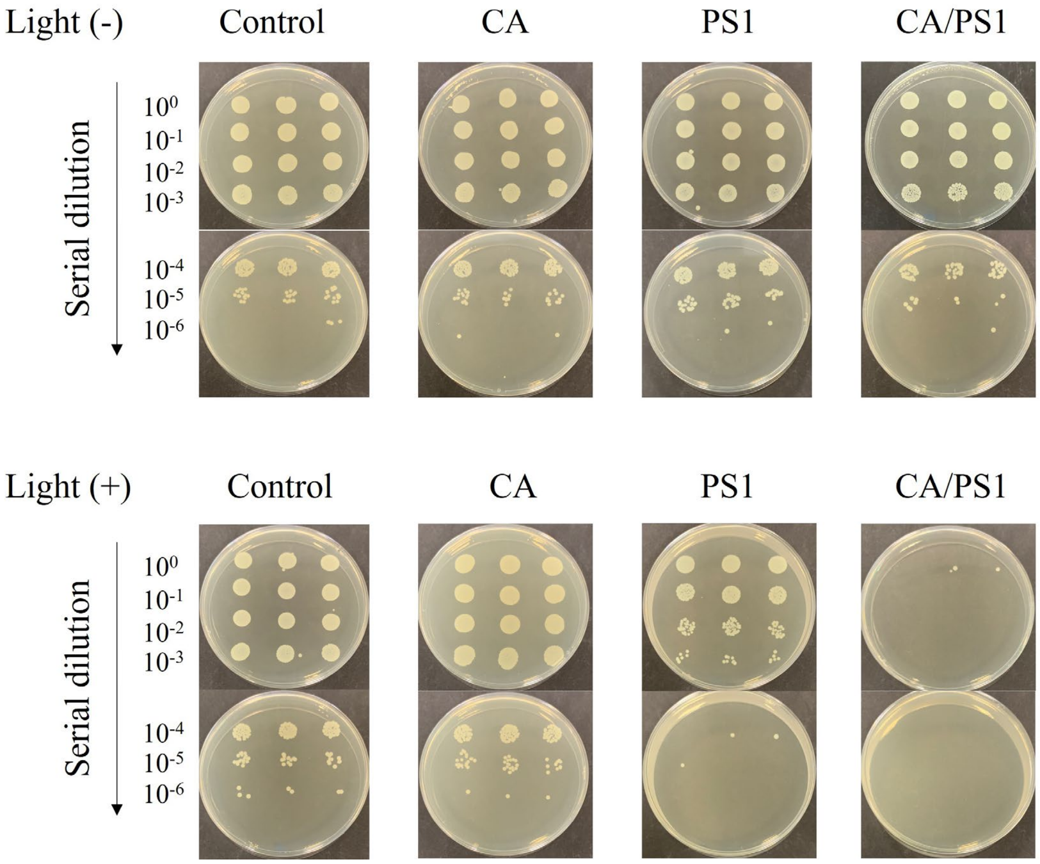

Supplementary Figure 9. Photographs of the LB agar plates after the inoculation and overnight incubation of *S. aureus* cultures treated with CA/PS1 (PS1 4 μg/mL, CA 150 μg/mL), PS1 (4 μg/mL), or CA (150 μg/mL) under normoxic conditions with or without light irradiation (60 mW/cm^2, 10 min).

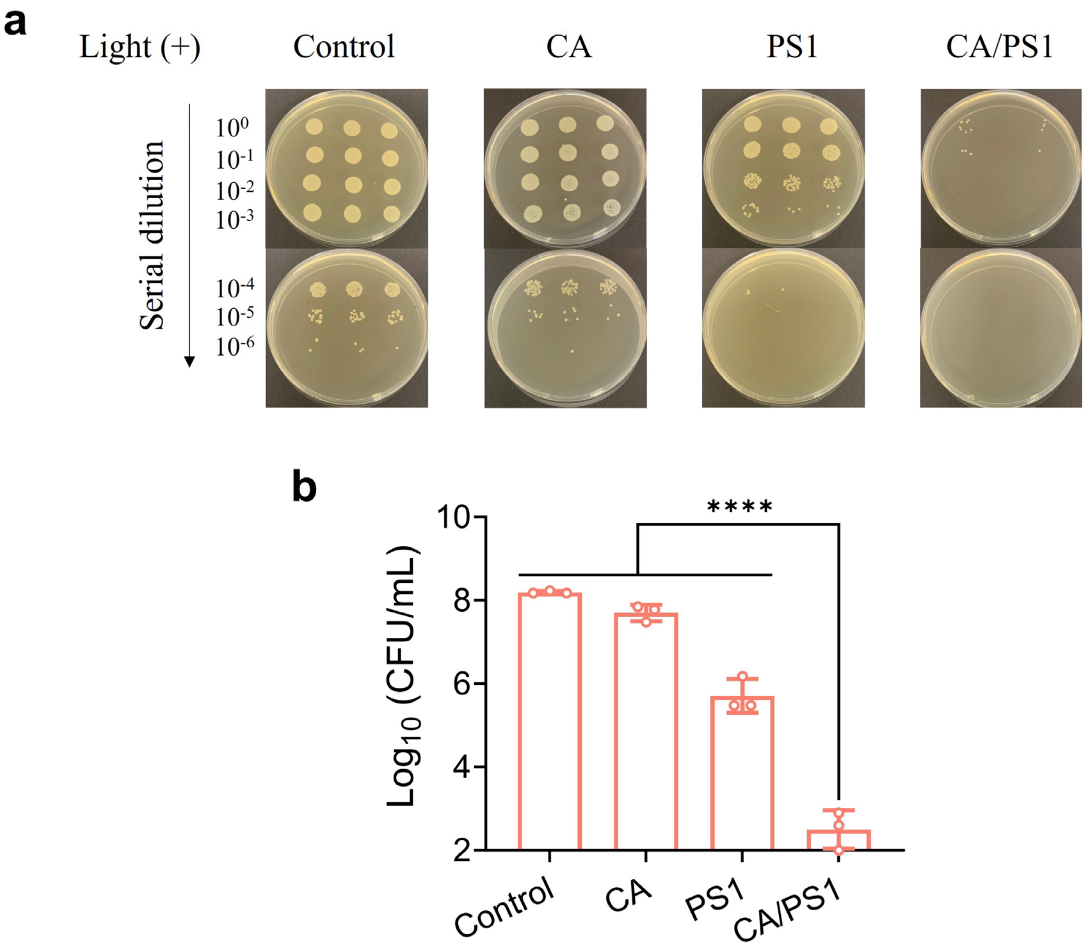

Supplementary Figure 10. (a) Photographs of the LB agar plates after the inoculation and overnight incubation of methicillin-resistant *Staphylococcus aureus* (MRSA) cultures treated with CA/PS1 (PS1 4 μg/mL, CA 150 μg/mL), PS1 (4 μg/mL), or CA (150 μg/mL) with or without light irradiation (60 mW/cm^2, 10 min). (b) CFU counts of MRSA from the antibacterial assay shown in a. Data represented as mean ± SD from n = 3 independent biological samples. Statistical significance was analyzed via one-way ANOVA test with a Tukey post hoc test, ****$P < 0.0001$.

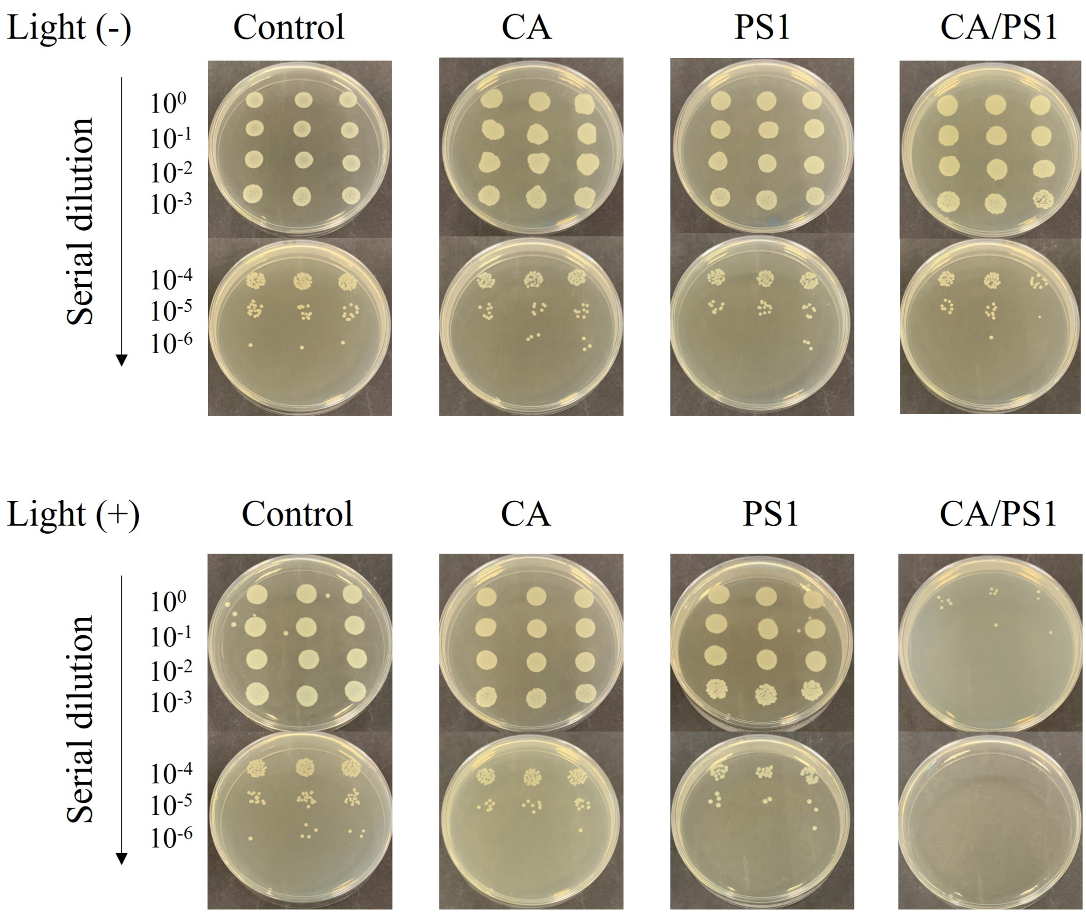

Supplementary Figure 11. Photographs of the LB agar plates after the inoculation and overnight incubation of *S. aureus* cultures treated with CA/PS1 (PS1 4 μg/mL, CA 150 μg/mL), PS1 (4 μg/mL), or CA (150 μg/mL) under hypoxic conditions with or without light irradiation (60 mW/cm^2, 10 min).

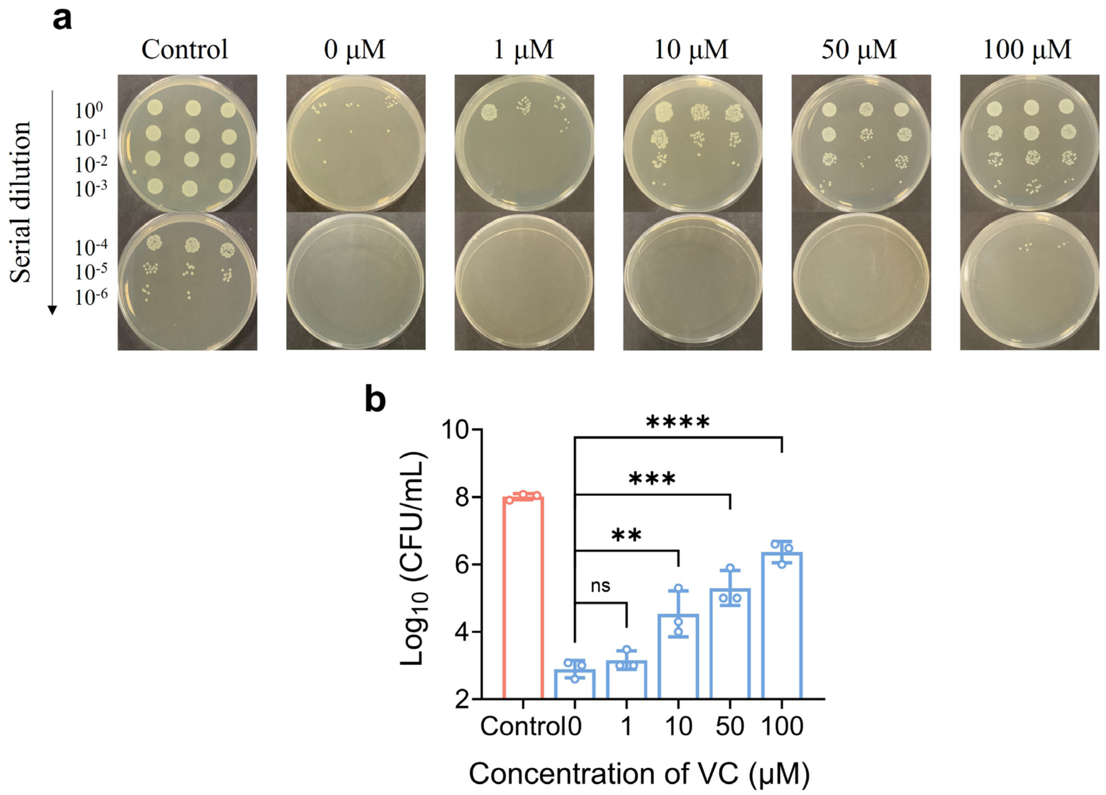

Supplementary Figure 12. (a) Photographs of the LB agar plates after the inoculation and overnight incubation of *S. aureus* cultures treated with the addition of VC (0-100 μM) upon light irradiation (60 mW/cm^2, 10 min) in the presence of CA/PS1 (PS1 4 μg/mL, CA 150 μg/mL). (b) CFU counts of *S. aureus* from the antibacterial assay shown in a. Data represented as mean ± SD from n = 3 independent biological samples. Statistical significance was analyzed via one-way ANOVA test with a Tukey post hoc test, **P < 0.01, ***P < 0.001, and ****P < 0.0001. ns, no significance.

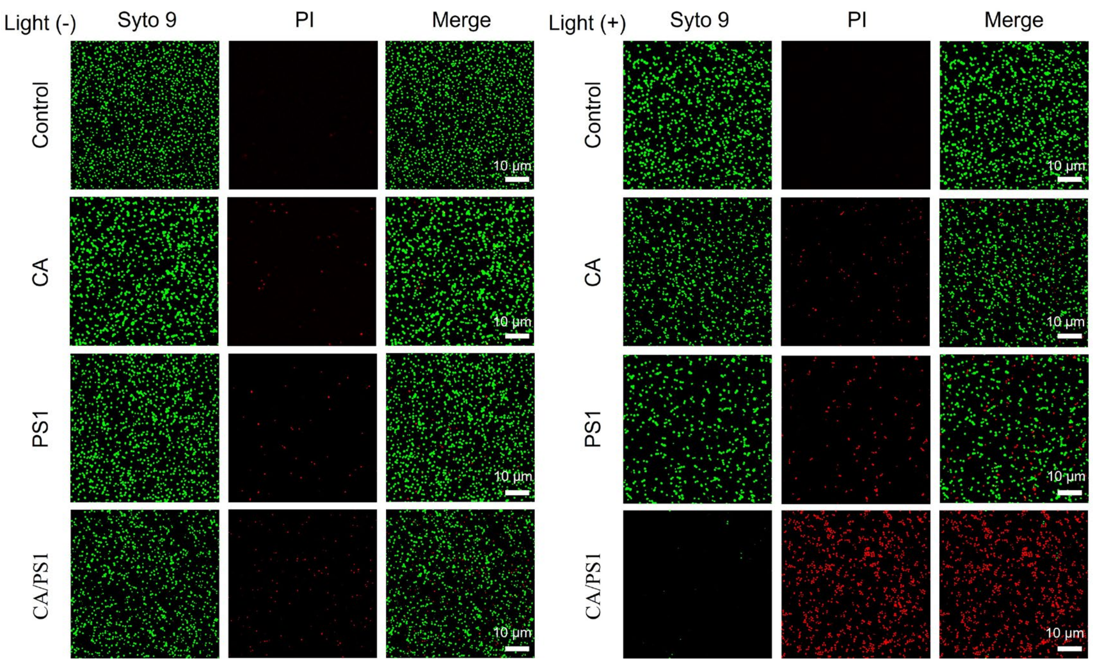

Supplementary Figure 13. Representative live/dead bacterial staining images of *S. aureus* treated with or without light irradiation in the presence of CA/PS1 (PS1 4 µg/mL, CA 150 µg/mL), PS1 (4 µg/mL), or CA (150 µg/mL). Green fluorescence: live bacteria, red fluorescence: dead bacteria. Scale bar = 10 µm.

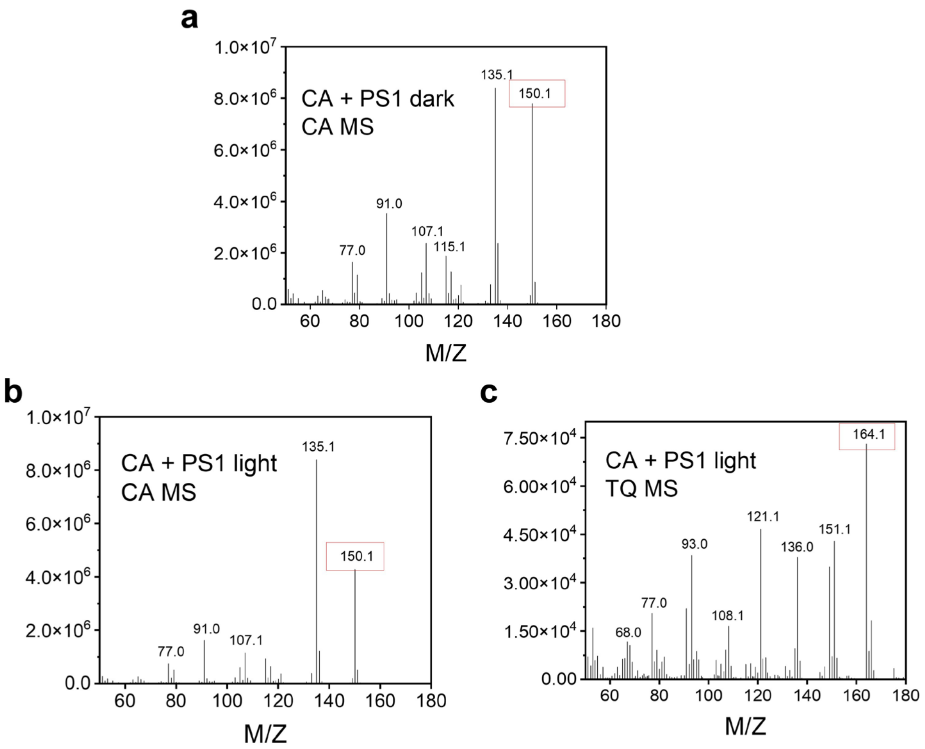

Supplementary Figure 14. The corresponding mass spectra of GC-MS analysis of CA mixed with PS1 when treated without (a) or with (b)-(c) light irradiation.

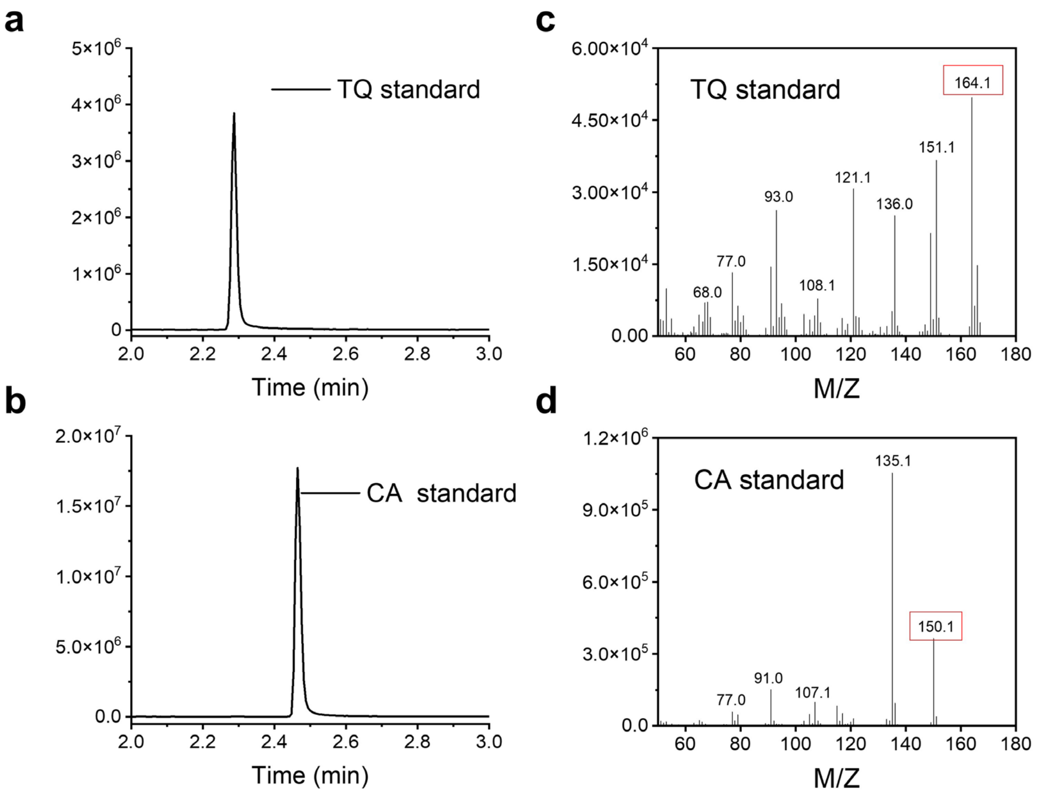

Supplementary Figure 15. GC-MS analysis of TQ (a) and CA (b) standards. The retention time of TQ and CA are 2.28 min and 2.47 min, respectively. The corresponding mass spectra of TQ (c) and CA (d).

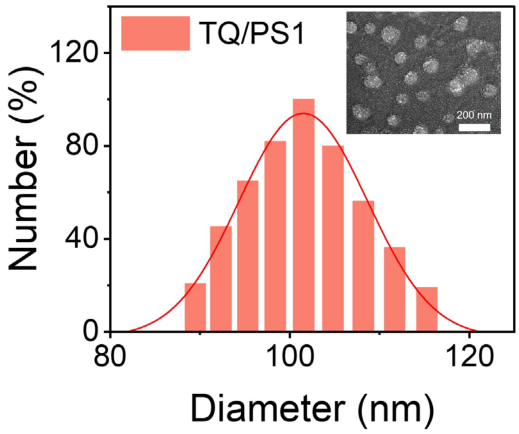

Supplementary Figure 16. Hydrodynamic particle diameter measured by DLS of TQ/PS1 complex in PBS, insert is the SEM image of TQ/PS1 complex. Scale bar = 200 nm.

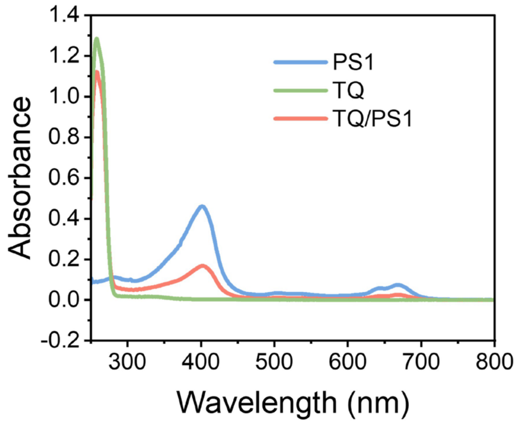

Supplementary Figure 17. UV-Vis spectra of PS1, TQ, and TQ/PS1. The samples were filtered through a 0.45 μm filter and then characterized by UV-Vis.

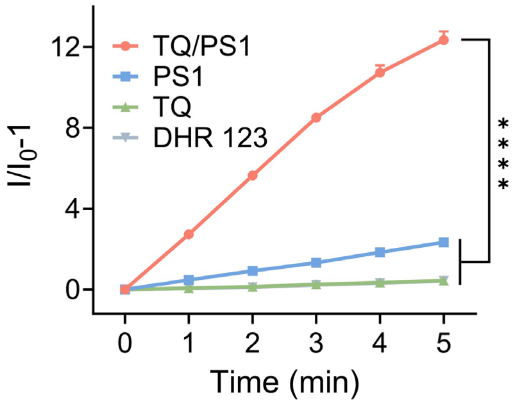

Supplementary Figure 18. Comparison of the PL intensity of DHR 123 alone and DHR 123 treated with TQ/PS1 (PS1 4 μg/mL, TQ 10 μg/mL), PS1 (4 μg/mL), or TQ (10 μg/mL) under light irradiation for $O_2^{-\bullet}$ detection. Data presented as mean ± SD derived from n = 3 independent samples. Statistical significance was analyzed via one-way ANOVA test with a Tukey post hoc test, ****$P < 0.0001$.

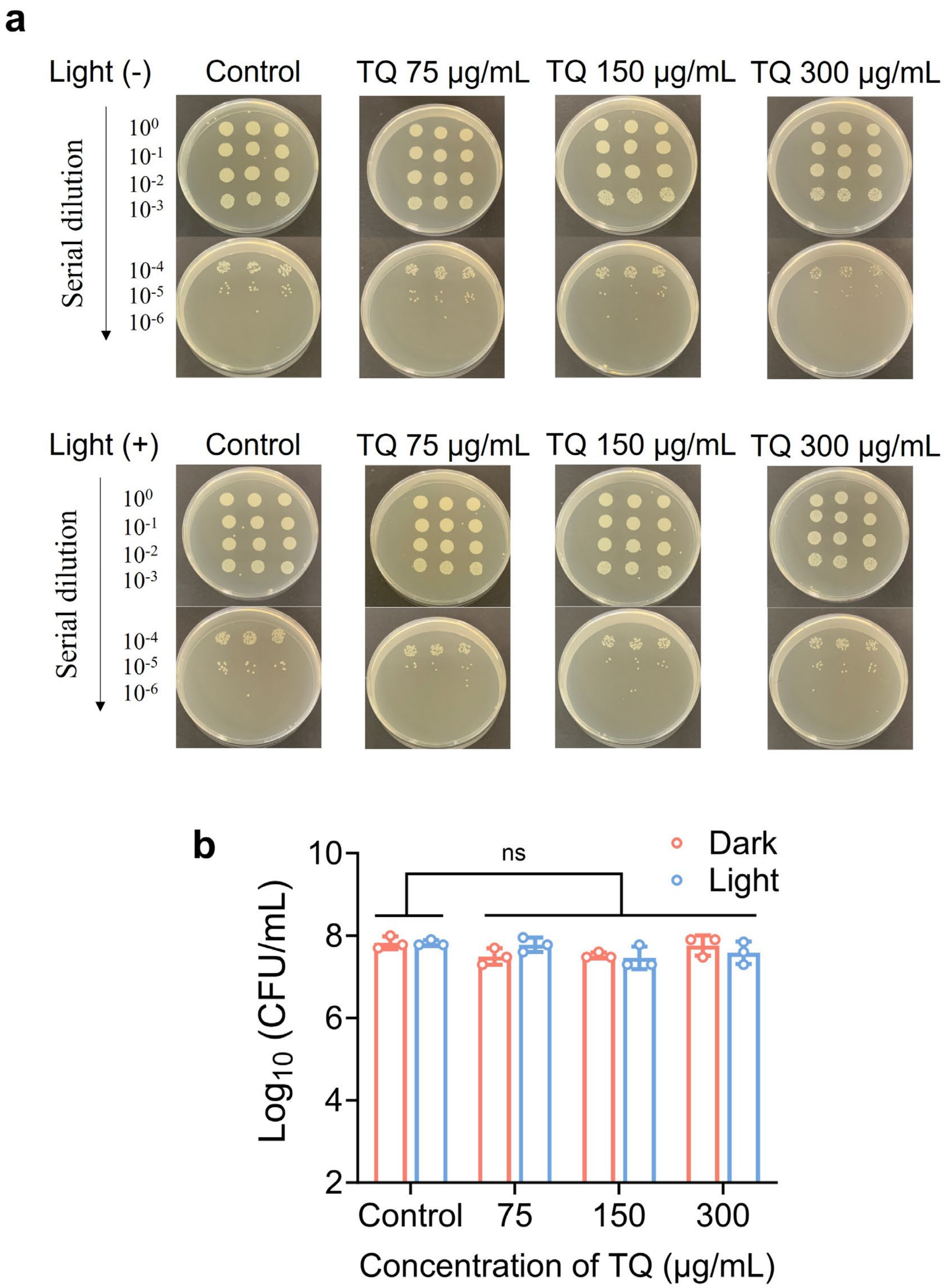

Supplementary Figure 19. (a) Photographs of the LB agar plates after the inoculation and overnight incubation of *S. aureus* cultures treated with different concentrations of TQ (0-300 μg/mL) with or without light irradiation (60 mW/cm^2, 10 min) (b) CFU counts from the antibacterial assay shown in a. Data represented as mean ± SD from n = 3 independent biological samples. Statistical significance was analyzed via two-way ANOVA test with a Tukey post hoc test. ns, no significance.

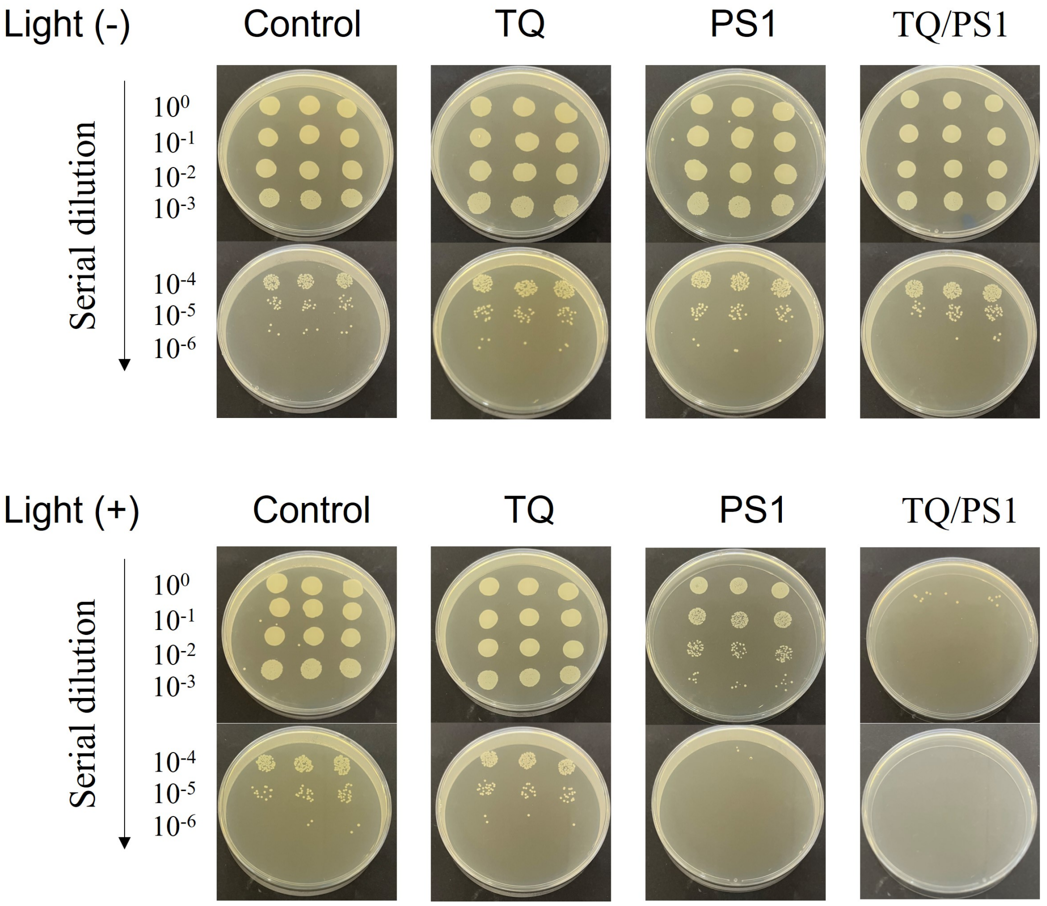

Supplementary Figure 20. Photographs of the LB agar plates after the inoculation and overnight incubation of *S. aureus* cultures treated with or without light irradiation (60 mW/cm^2, 10 min) in the presence of TQ/PS1 (PS1 4 μg/mL, TQ 8 μg/mL), PS1 (4 μg/mL), or TQ (8 μg/mL).

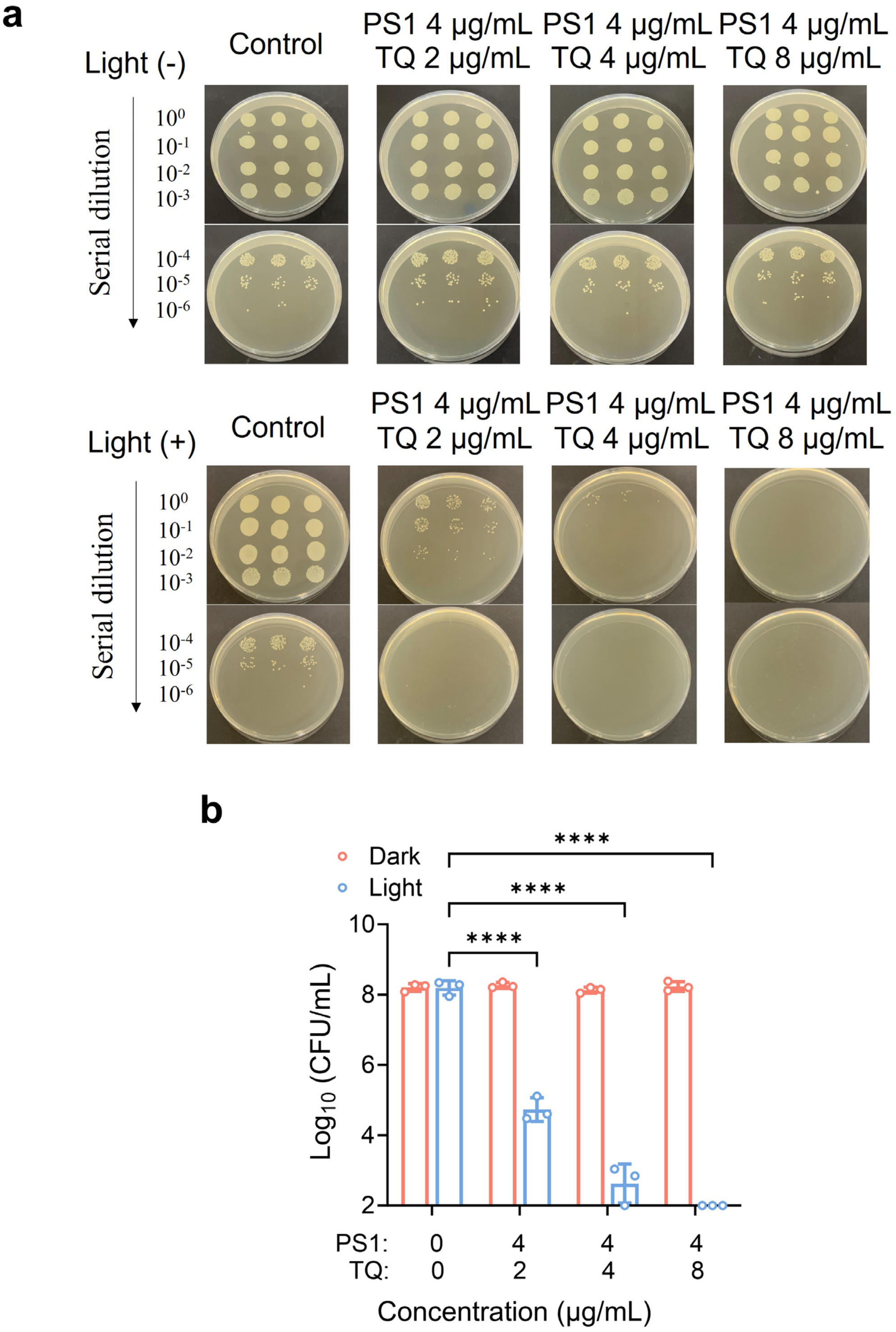

Supplementary Figure 21. (a) Photographs of the LB agar plates after the inoculation and overnight incubation of *S. aureus* cultures treated with different concentrations of TQ/PS1 complex with or without light irradiation (60 mW/cm^2, 10 min) (b) CFU counts of *S. aureus* from the antibacterial assay shown in a. Data represented as mean ± SD from n = 3 independent biological samples. Statistical significance was analyzed via two-way ANOVA test with a Tukey post hoc test. ****P < 0.0001.

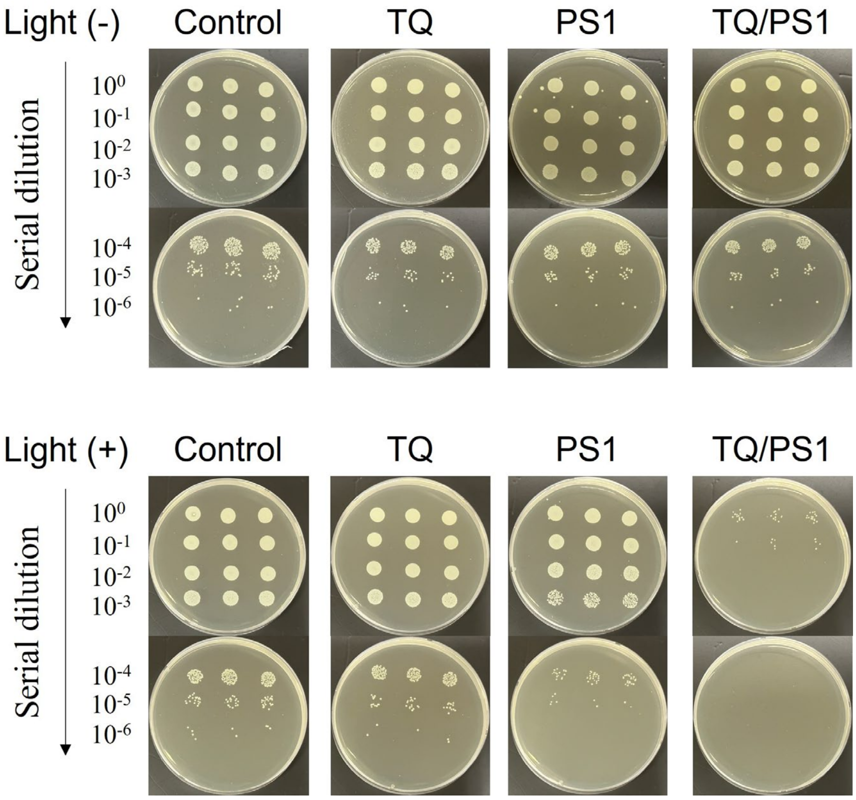

Supplementary Figure 22. Photographs of the LB agar plates after the inoculation and overnight incubation of *S. aureus* cultures treated with or without light irradiation (60 mW/cm^2, 10 min) under hypoxic conditions in the presence of TQ/PS1 (PS1 4 μg/mL, TQ 8 μg/mL), PS1 (4 μg/mL), or TQ (8 μg/mL).

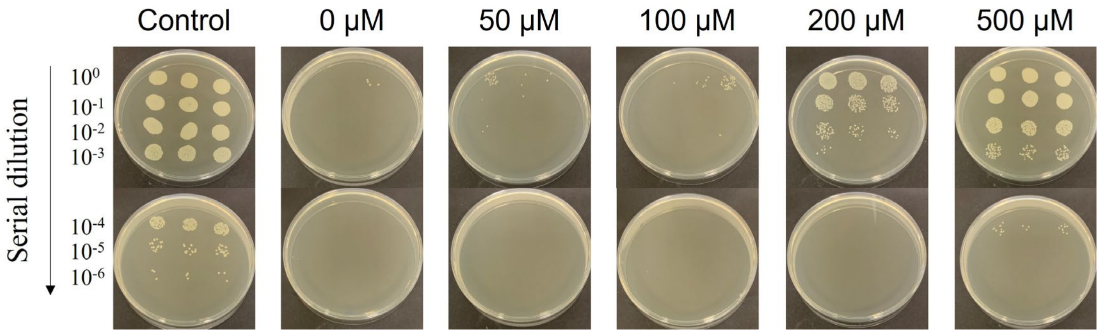

Supplementary Figure 23. Photographs of the LB agar plates after the inoculation and overnight incubation of *S. aureus* cultures treated with the addition of VC (0-500 μM) in the presence of TQ/PS1 complex (PS1 4 μg/mL, TQ 8 μg/mL) upon light irradiation (60 mW/cm^2, 10 min).

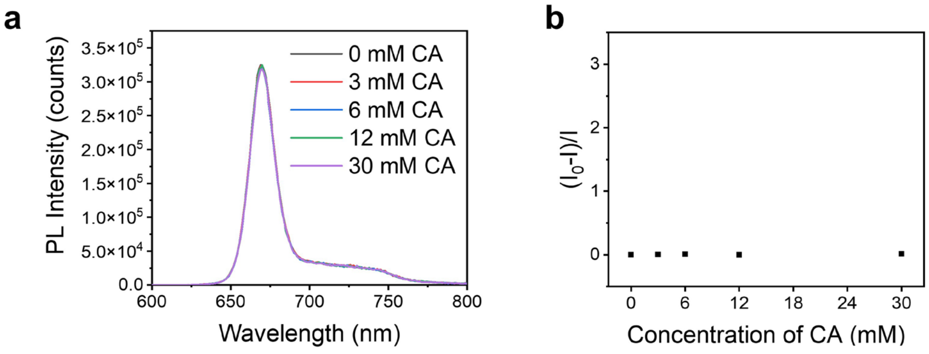

Supplementary Figure 24. (a) The emission spectra change of PS1 (10 μM) with increasing concentrations of CA (0-30 mM) under excitation at 405 nm in DMF. (b) Stern-Volmer plot of fluorescence intensity change of PS1 against different concentrations of CA in DMF. Where I_0 is the fluorescence intensity in the absence of CA; I is the fluorescence intensity of PS1 in the presence of different concentrations of CA.

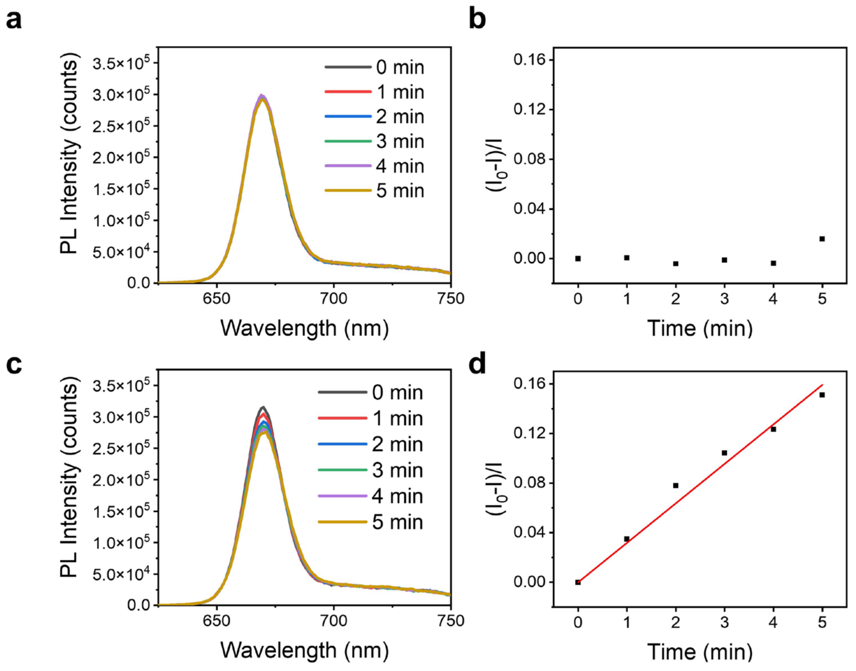

Supplementary Figure 25. (a) The emission spectra change of PS1 (10 μM) upon light irradiation in different light irradiation time under excitation at 405 nm in DMF. (b) Plot of fluorescence intensity change of PS1 against different light irradiation time in DMF. Where I_0 is the fluorescence intensity without light irradiation; I is the fluorescence intensity of PS1 upon different light irradiation time. (c) The emission spectra change of PS1 (10 μM) treated with CA upon different light irradiation time under excitation at 405 nm in DMF. (d) Plot of fluorescence intensity change of PS1 against different light irradiation time in DMF. Where I_0 is the fluorescence intensity treated with CA without light irradiation; I is the fluorescence intensity of PS1 treated with CA upon different light irradiation time.

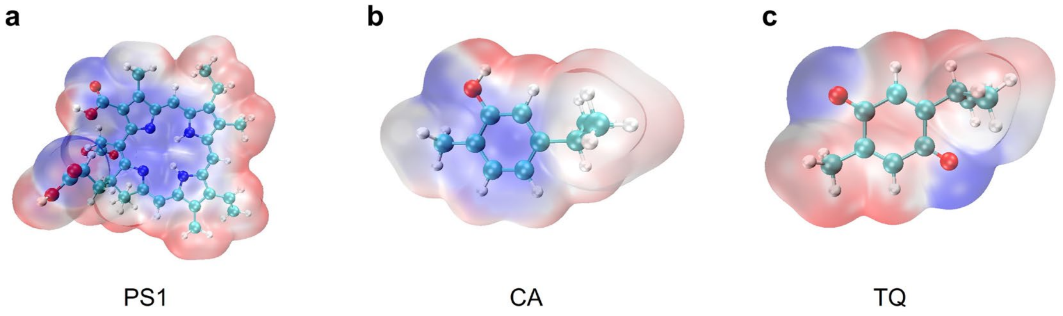

Supplementary Figure 26. Simulated electrostatic potential maps of PS1 (a), CA (b), TQ (c).

Supplementary Table 1. Calculated energy and adsorption energy.

	E (Hartree)	Adsorption energy (Hartree)
PS1	-1987.02	-
CA	-464.655	-
TQ	-538.641	-
CA/PS1	-2452.46	-0.77911
TQ/PS1	-2526.45	-0.078796

1 Hartree = 2625.5 kJ mol^{-1}

$$E_{ads} = E_{(ad+sub)} - E_{ad} - E_{sub} \quad (1)$$

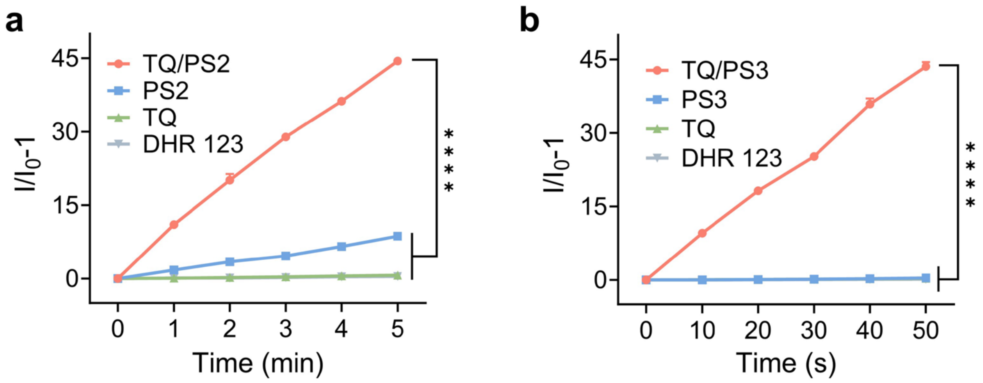

Supplementary Figure 27. (a) Comparison of the PL intensity of DHR 123 alone and DHR 123 treated with TQ/PS2 (PS2 4 µg/mL, TQ 10 µg/mL) or PS2 (4 µg/mL) under light irradiation for $O_2^{-\bullet}$ detection. (b) Comparison of the PL intensity of DHR 123 alone and DHR 123 treated with TQ/PS3 (PS3 4 µg/mL, TQ 10 µg/mL) or PS3 (4 µg/mL) under light irradiation for $O_2^{-\bullet}$ detection. Data in (a-b) are presented as mean ± SD derived from n = 3 independent samples. Statistical significance was analyzed via one-way ANOVA test with a Tukey post hoc test, ****P < 0.0001.

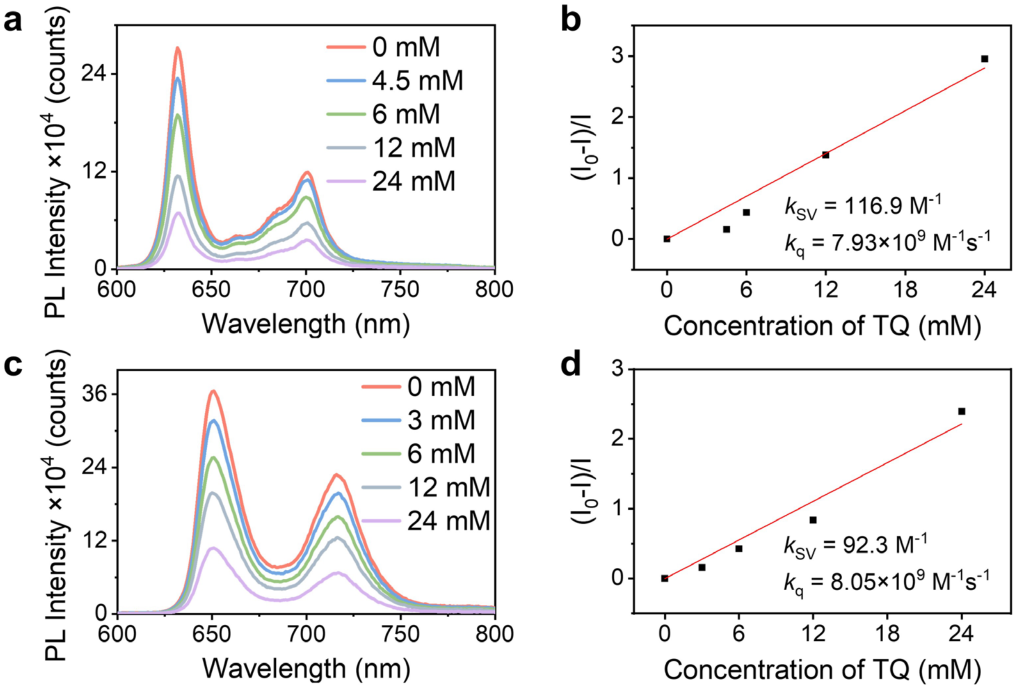

Supplementary Figure 28. The emission spectra change of PS2 (a) and PS3 (c) (10 µM) with increasing concentrations of TQ (0-24 mM) under excitation at 405 nm in DMF. Stern-Volmer plot of fluorescence intensity change of PS2 (b) and PS3 (d) against TQ in DMF. Where I_0 is the fluorescence intensity in the absence of TQ; I is the fluorescence intensity of PS in the presence of different concentrations of TQ.

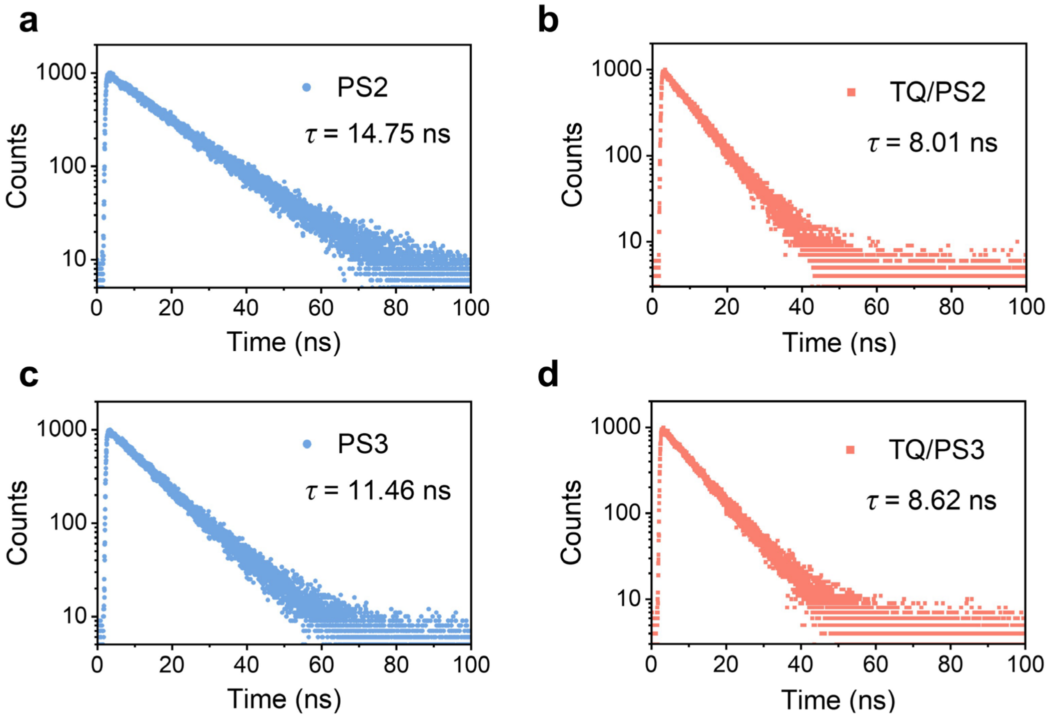

Supplementary Figure 29. Fluorescence decay curves of PS2 (a), TQ/PS2 (b) at peak of 632 nm. Fluorescence decay curves of PS3 (c), TQ/PS3 (d) at peak of 650 nm.

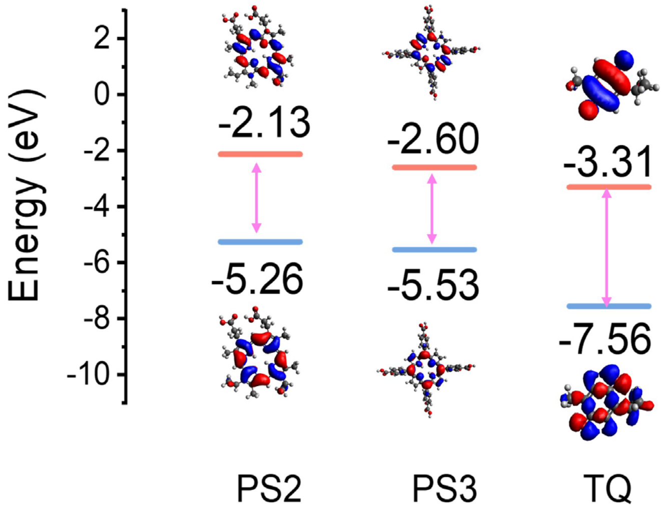

Supplementary Figure 30. HOMO and LUMO energy levels of PS2, PS3, and TQ.

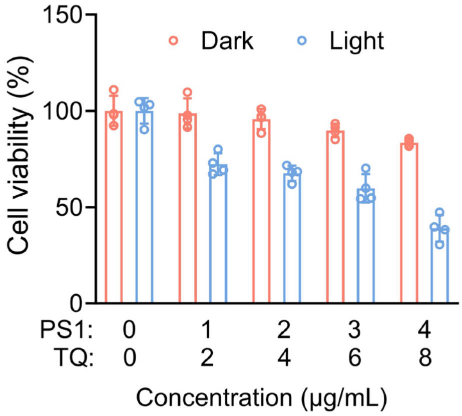

Supplementary Figure 31. Cell viability of NIH 3T3 fibroblast cells treated with different concentrations of TQ/PS1 complex in the presence and absence of light irradiation (60 mW/cm^2, 10 min) using MTT assay. Data presented as mean ± SD derived from n = 4 independent biological samples.

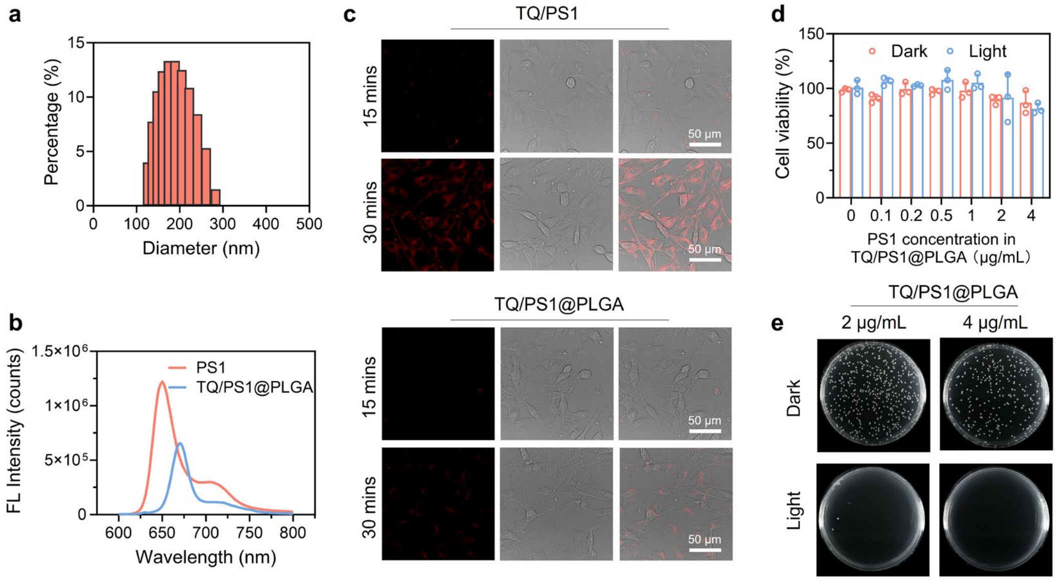

Supplementary Figure 32. (a) Diameter of TQ/PS1@PLGA measured by DLS. (b) Fluorescence of TQ/PS1@PLGA and PS1. (c) Fluorescence imaging comparison of cellular uptake of TQ/PS1 and TQ/PS1@PLGA within 15 and 30 min by NIH 3T3 fibroblast cells. (d) Cell viability of NIH 3T3 fibroblast cells treated with different concentrations of TQ/PS1@PLGA in the presence and absence of light irradiation (60 mW/cm^2, 10 min) using MTT assay, n = 3 independent samples. (e) Photographs of the LB agar plates after the inoculation and overnight incubation of *S. aureus* cultures treated with different concentrations of TQ/PS1@PLGA in the presence and absence of light irradiation (60 mW/cm^2, 10 min).

In this study, we observed that cell cytotoxicity of TQ/PS1 complex remains evident at higher concentrations (Supplementary Figure 31), highlighting the need for enhanced specificity of PSs between cells and bacteria to improve biocompatibility. To address this issue, we utilized a strategy by formulating PLGA-PEG nanoparticles with PS1 and TQ (abbreviated as TQ/PS1@PLGA), based on a reported method[1]. This technique can produce PS particles with a diameter of approximately 200 nm (Supplementary Figure 32. a). Fluorescence spectra analysis of TQ/PS1@PLGA confirmed successful encapsulation of TQ/PS1 within the particles (Supplementary Figure 32. b). At same incubation times and PS concentrations, the cellular fluorescence intensity after incubating with TQ/PS1@PLGA was significantly lower than that after incubating with TQ/PS1, demonstrating that this nanoparticle strategy significantly reduces cellular uptake of the PS (Supplementary Figure 32. c). This reduction is beneficial as it allows TQ/PS1@PLGA to maintain strong antibacterial efficacy while largely preserving the survival of fibroblast cells. (Supplementary Figure 32. d and e). These findings suggest that our developed PS system has considerable potential for further optimization to enhance biocompatibility and achieve better therapeutic outcomes.

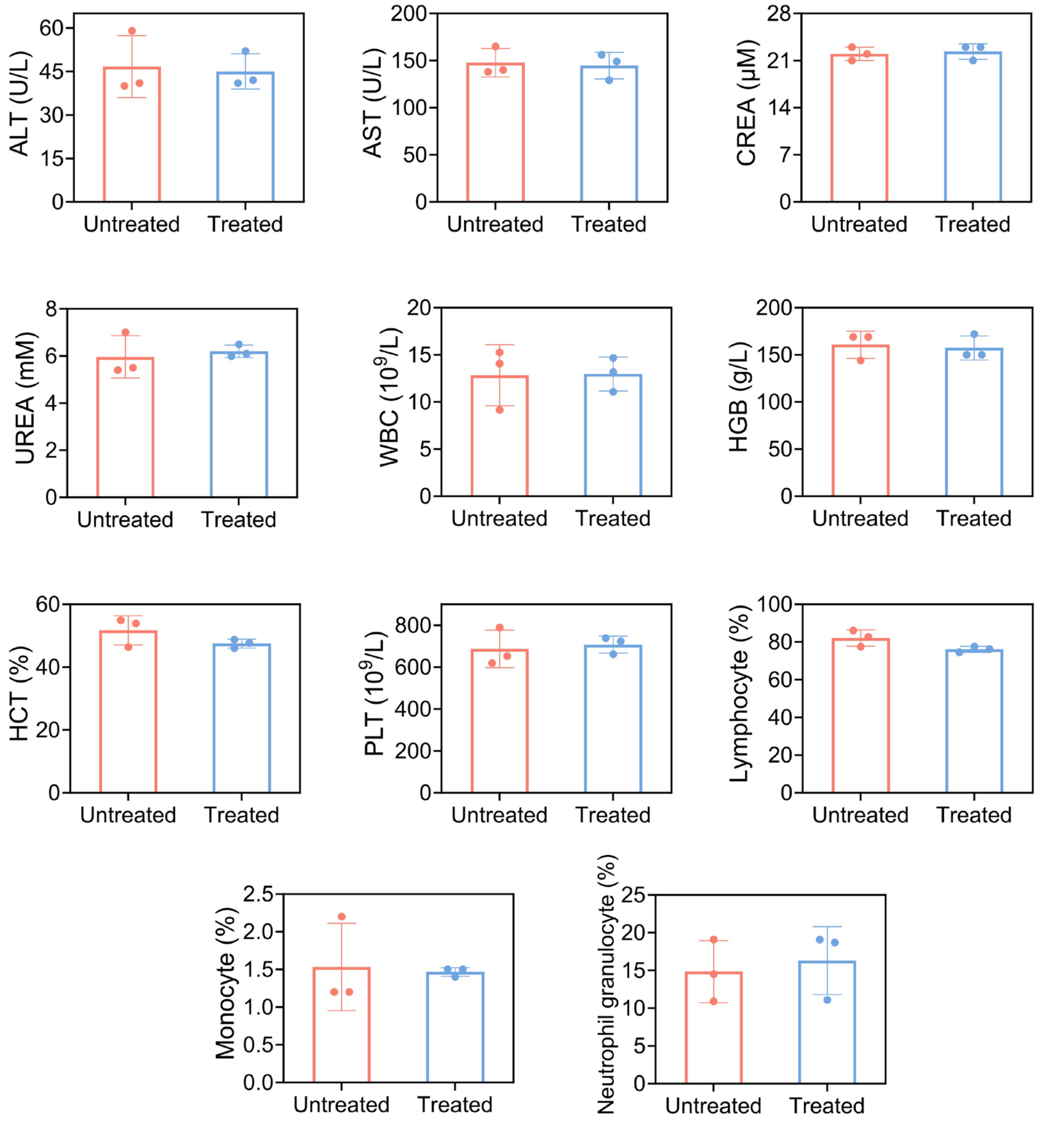

Supplementary Figure 33. Hematology and blood biochemistry test at 7 d after mice treated with TQ/PS1 complex, with untreated mice serving as the control group. Biochemical and hematological parameters include alanine aminotransferase (ALT), aspartate aminotransferase (AST), creatinine (CREA), urea (UREA), white blood cell count (WBC), hemoglobin (HGB), hematocrit (HCT), platelet count (PLT), and the percentages of immune cells, specifically lymphocytes, monocytes, and neutrophil granulocytes. Data presented as mean ± SD derived from n = 3 independent biological samples.

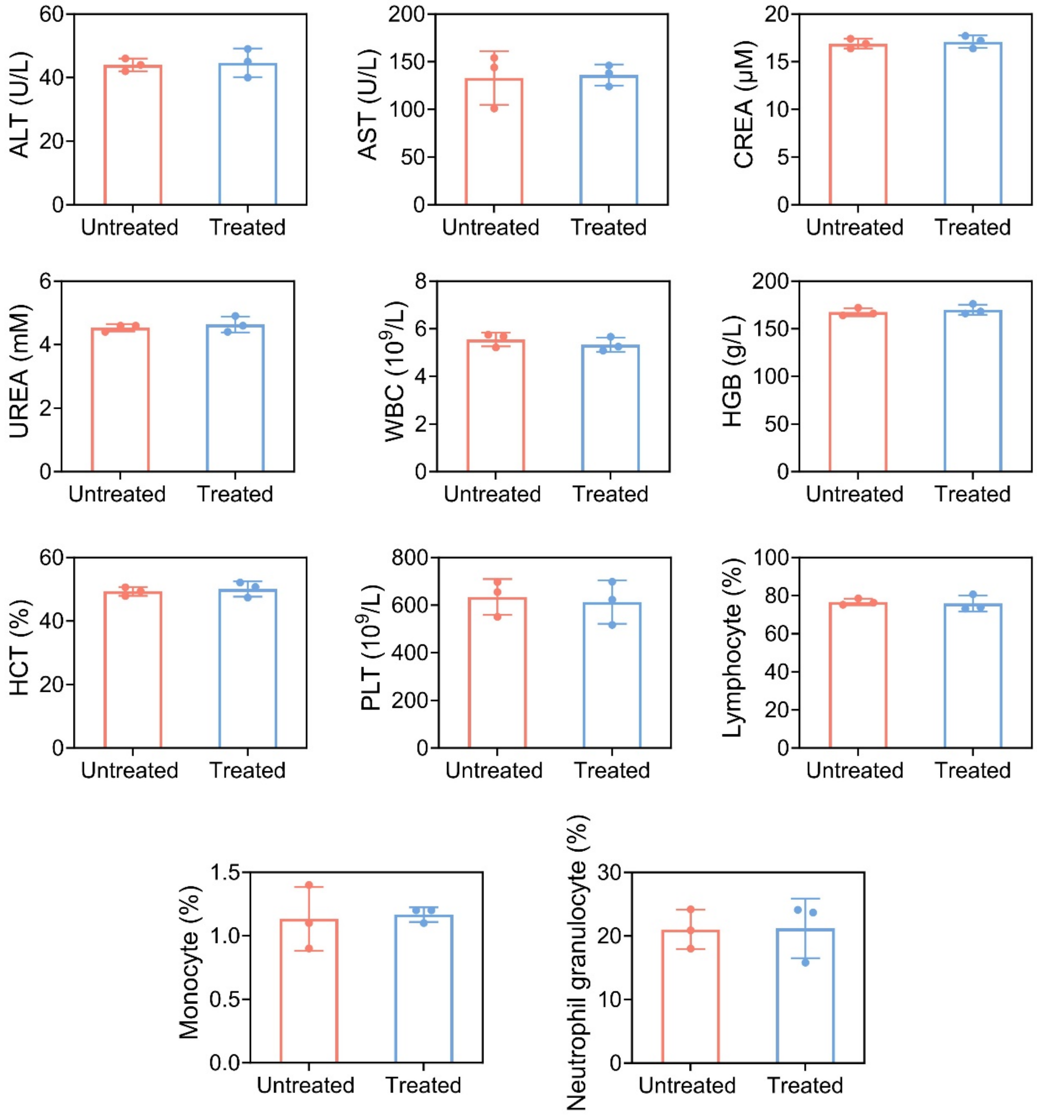

Supplementary Figure 34. Hematology and blood biochemistry test at 1 d after the mice treated with TQ/PS1 complex, with untreated mice serving as the control group. The untreated mice served as the control group. The analysis included biochemical indicators such as alanine aminotransferase (ALT), aspartate aminotransferase (AST), creatinine (CREA), urea (UREA), white blood cell count (WBC), hemoglobin (HGB), hematocrit (HCT), platelet count (PLT), and the percentages of immune cells, specifically lymphocytes, monocytes, and neutrophil granulocytes. Data presented as mean ± SD derived from n = 3 independent biological samples.

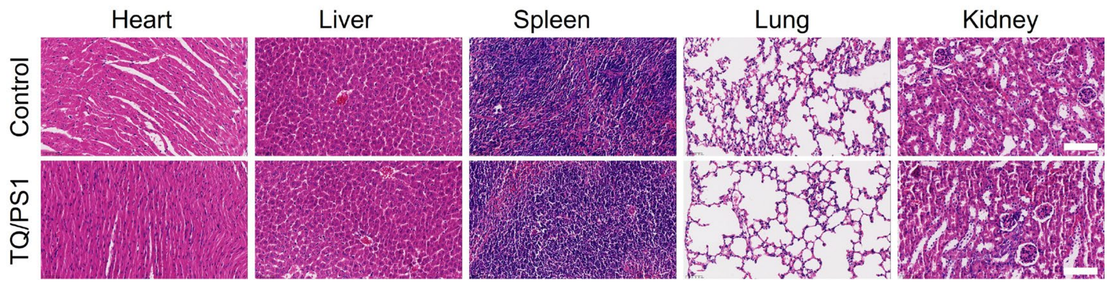

Supplementary Figure 35. Histological images of major organs, including heart, liver, spleen, lung, and kidney, at 7 d after mice treated with TQ/PS1 complex, with untreated mice serving as the control group. Scale bar = 100 μm.

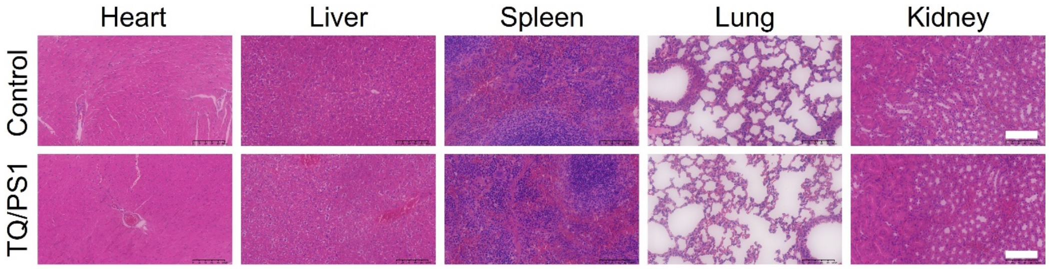

Supplementary Figure 36. Histological images of major organs, including heart, liver, spleen, lung, and kidney, at 1 d after mice treated with TQ/PS1 complex, with untreated mice serving as the control group. Scale bar = 100 μm.

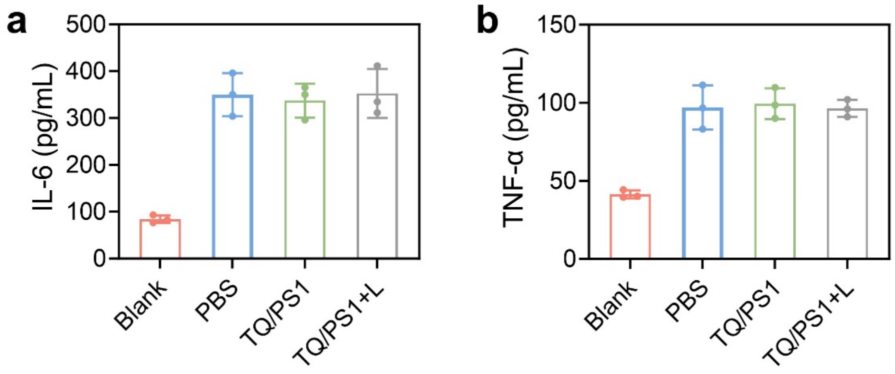

Supplementary Figure 37. Secretion levels of IL-6 (a) and TNF-α (b) collected from tissues of open wounds were measured at 1 d post-treatment, with healthy mice serving as blank control. Data presented as mean ± SD derived from n = 3 independent biological samples.

Supplementary References

1. Zhou Y, Wu W, Yang P, Mao D, Liu B. Near-infrared chemiluminescent nanoprobes for deep imaging and synergistic photothermal-nitric-oxide therapy of bacterial infection. *Biomaterials* **288**, 121693 (2022).
