# Supplementary material for: Thymoquinone as an electron transfer mediator to convert Type II photosensitizers to Type I photosensitizers
Source: Nat Commun. 2024 Jun 10;15:4943. doi: 10.1038/s41467-024-49311-z (PMC11164902; doi:10.1038/s41467-024-49311-z)
Supplement: Supplementary file 2 — Reporting Summary [file 41467_2024_49311_MOESM2_ESM.pdf]

nature portfolio

Corresponding author(s): Duo Mao, Bin Liu

Last updated by author(s): May 14, 2024

Reporting Summary

Nature Portfolio wishes to improve the reproducibility of the work that we publish. This form provides structure for consistency and transparency in reporting. For further information on Nature Portfolio policies, see our Editorial Policies and the Editorial Policy Checklist.

Statistics

For all statistical analyses, confirm that the following items are present in the figure legend, table legend, main text, or Methods section.

n/a	Confirmed	
☐	☒	The exact sample size (*n*) for each experimental group/condition, given as a discrete number and unit of measurement
☐	☒	A statement on whether measurements were taken from distinct samples or whether the same sample was measured repeatedly
☐	☒	The statistical test(s) used AND whether they are one- or two-sided<br>*Only common tests should be described solely by name; describe more complex techniques in the Methods section.*
☒	☐	A description of all covariates tested
☒	☐	A description of any assumptions or corrections, such as tests of normality and adjustment for multiple comparisons
☐	☒	A full description of the statistical parameters including central tendency (e.g. means) or other basic estimates (e.g. regression coefficient) AND variation (e.g. standard deviation) or associated estimates of uncertainty (e.g. confidence intervals)
☐	☒	For null hypothesis testing, the test statistic (e.g. *F*, *t*, *r*) with confidence intervals, effect sizes, degrees of freedom and *P* value noted<br>*Give P values as exact values whenever suitable.*
☒	☐	For Bayesian analysis, information on the choice of priors and Markov chain Monte Carlo settings
☒	☐	For hierarchical and complex designs, identification of the appropriate level for tests and full reporting of outcomes
☒	☐	Estimates of effect sizes (e.g. Cohen's *d*, Pearson's *r*), indicating how they were calculated

Our web collection on statistics for biologists contains articles on many of the points above.

Software and code

Policy information about availability of computer code

Data collection	The PL lifetime measurements were carried out by Edinburgh Instruments FLS 1000. The photoluminescence (PL) spectra were characterized by the Perkin-Elmer LS 55 spectrometer and infinite M200 PRO microplate reader. The absorption of samples was performed on a Shimadzu UV-1700 ultraviolet-visible (UV-vis) spectrophotometer. Hydrodynamic size distributions were measured on a Malvern Instruments Zetasizer Nano ZS ZEN3600 analyzer at 25 °C. The scanning electron microscope (SEM) was recorded by JEOL JSM-7610F Plus. Confocal images were obtained by Leica SP8 confocal laser scanning microscope (CLSM). GC-MS analysis was conducted by Agilent Technologies 7890B GC system. Electrochemical experiments were conducted by ATUOLAB PGSTAT302N. Light irradiation was conducted using an L-150A-1 single optical fiber light source (Microscope X). DFT calculations was conducted by Gaussian/16.C.02-AVX2.
Data analysis	All statistical analyses were carried out by using Graphpad Prism (version 10.0.0) and Origin 2021.

For manuscripts utilizing custom algorithms or software that are central to the research but not yet described in published literature, software must be made available to editors and reviewers. We strongly encourage code deposition in a community repository (e.g. GitHub). See the Nature Portfolio guidelines for submitting code & software for further information.

Data

Policy information about availability of data

All manuscripts must include a data availability statement. This statement should provide the following information, where applicable:
- Accession codes, unique identifiers, or web links for publicly available datasets
- A description of any restrictions on data availability
- For clinical datasets or third party data, please ensure that the statement adheres to our policy

The data generated in this study are available within the article, Supplementary Information, and Source Data. Source data are provided with this paper.

Research involving human participants, their data, or biological material

Policy information about studies with human participants or human data. See also policy information about sex, gender (identity/presentation), and sexual orientation and race, ethnicity and racism.

Reporting on sex and gender	N/A
Reporting on race, ethnicity, or other socially relevant groupings	N/A
Population characteristics	N/A
Recruitment	N/A
Ethics oversight	N/A

Note that full information on the approval of the study protocol must also be provided in the manuscript.

Field-specific reporting

Please select the one below that is the best fit for your research. If you are not sure, read the appropriate sections before making your selection.

- [x] Life sciences
- [ ] Behavioural & social sciences
- [ ] Ecological, evolutionary & environmental sciences

For a reference copy of the document with all sections, see nature.com/documents/nr-reporting-summary-flat.pdf

Life sciences study design

All studies must disclose on these points even when the disclosure is negative.

Sample size	The sample size was according to the pilot study and standard protocols. The sample size (n) of each experiment is provided in the corresponding figure caption.
Data exclusions	No data were excluded.
Replication	The experiments were repeated with n = 3-5 independent samples and exact number of n is provided in the figure caption.
Randomization	The mice were randomly distributed into different group for experimental purpose.
Blinding	The investigators were kept unaware of the data collection and analysis during experiments.

Reporting for specific materials, systems and methods

We require information from authors about some types of materials, experimental systems and methods used in many studies. Here, indicate whether each material, system or method listed is relevant to your study. If you are not sure if a list item applies to your research, read the appropriate section before selecting a response.

Materials & experimental systems

n/a	Involved in the study
☐	☒ Antibodies
☐	☒ Eukaryotic cell lines
☒	☐ Palaeontology and archaeology
☐	☒ Animals and other organisms
☒	☐ Clinical data
☒	☐ Dual use research of concern
☐	☒ Plants

Methods

n/a	Involved in the study
☒	☐ ChIP-seq
☒	☐ Flow cytometry
☒	☐ MRI-based neuroimaging

Antibodies

Antibodies used	TNF-α ELISA kit (m1002095N) and IL-6 ELISA kit (ml098430)
Validation	The kits are commercially available and validated by the manufacturers, as indicated on the website of supplier, please refer to the corresponding website for more details.

Eukaryotic cell lines

Policy information about cell lines and Sex and Gender in Research

Cell line source(s)	NIH/3T3 fibroblast Cells were purchased from ATCC.
Authentication	The cell line used in this study was not authenticated by the authors.
Mycoplasma contamination	No mycoplasma contamination was found.
Commonly misidentified lines (See ICLAC register)	No commonly misidentified cell lines were used.

Animals and other research organisms

Policy information about studies involving animals; ARRIVE guidelines recommended for reporting animal research, and Sex and Gender in Research

Laboratory animals	Female BALB/c mice (6-8 weeks old) were purchased from Zhuhai Baitantong Biotechnology Co., Ltd. All mice used in this study were maintained in a dedicated pathogen-free animal facility at 60% of humidity at degrees Celsius with 12/12 light schedule and free access to food and water.
Wild animals	The study did not involve wild animals.
Reporting on sex	Female BALB/c mice (6-8 weeks old) were used in this study. Sex was not considering in this study.
Field-collected samples	The study does not involve field-collected study.
Ethics oversight	All animal studies were performed in accordance with the guidelines set by the Guangdong Huawei Testing Co., Ltd.

Note that full information on the approval of the study protocol must also be provided in the manuscript.

Plants

Seed stocks	N/A
Novel plant genotypes	N/A
Authentication	N/A
